# Differential Effects of Twice vs. Four Times Weekly Combined Exercise Training in Aging Adults With Hypertension: A Randomized Clinical Trial

**DOI:** 10.1111/sms.70239

**Published:** 2026-03-06

**Authors:** Rodrigo Ferrari, Vinícius Mallmann Schneider, Lucas Betti Domingues, Rodrigo Abreu, Gabriel Lemes, Rodrigo Leal‐Menezes, Hirofumi Tanaka, Sandra Costa Fuchs, Leandro de Oliveira Carpes

**Affiliations:** ^1^ Postgraduate Program in Cardiology, School of Medicine Universidade Federal Do Rio Grande Do Sul Porto Alegre RS Brazil; ^2^ Sports and Exercise Training Study Group, Clinical Research Center Hospital De Clínicas De Porto Alegre RS Brazil; ^3^ Postgraduate Program in Human Movement Sciences, School of Physical Education Universidade Federal Do Rio Grande Do Sul Porto Alegre RS Brazil; ^4^ Department of Kinesiology and Health Education The University of Texas at Austin Austin Texas USA; ^5^ Faculty of Physical Education Universidade Do Extremo Sul Catarinense Criciúma SC Brazil

**Keywords:** aerobic exercise training, ambulatory (24‐h) blood pressure, physical activity, resistance training

## Abstract

Current exercise guidelines emphasize a total weekly volume of exercise (i.e., 150 min/week), but the optimal weekly frequency remains uncertain. Therefore, this randomized clinical trial aimed to compare the effects of combined resistance and aerobic training either two or four times per week, at the same total weekly volume, on office and ambulatory blood pressure in older adults with hypertension. Participants were randomized to exercise training performed either twice per week (CT2, *n* = 49) or four times per week (CT4, *n* = 49), for 12 weeks. Primary outcome was 24‐h ambulatory BP; secondary outcomes included office BP and physical fitness, assessed at baseline and post‐intervention. Of the 98 randomized participants (66% women; mean age 64 ± 7 years), all were included in the intention‐to‐treat analysis, whereas 63 were retained for the per‐protocol analysis after excluding those with low adherence (< 80%) or missing post‐intervention data. Although no significant differences between groups were observed in office and ambulatory BP in the intention‐to‐treat analysis, office systolic (−8 mmHg, *p* = 0.001) and diastolic BP (−3 mmHg, *p* = 0.001) reduced in CT4 (−8 mmHg, *p* = 0.001), and systolic BP decreased in CT2 after training (−4 mmHg, *p* = 0.032). In the per‐protocol analysis, office systolic BP was lower in CT4 than CT2 after training (−6 mmHg, *p* = 0.049). CT4 demonstrated significant reductions in 24‐h systolic (−4 mmHg, *p* = 0.012) and diastolic BP (−2 mmHg, *p* = 0.010) after training. Cardiorespiratory fitness improved in CT4 (Δ = 8%, *p* < 0.001) but not in CT2. Our findings suggest that higher weekly frequency may optimize blood pressure management in hypertension, although these additional benefits appear to be dependent on high protocol adherence.

**Trial Registration:** ClinicalTrials.gov Identifier: NCT04218903

## 1 Introduction

Hypertension has been and remains a leading modifiable risk factor for cardiovascular disease and mortality, with its prevalence increasing markedly over the lifespan [1]. Although the management of hypertension commonly involves antihypertensive medications, lifestyle interventions in general and regular exercise training in particular are the first‐line therapeutic strategy for the management of hypertension [2].

The American College of Sports Medicine, the American Heart Association, and other organizations have provided exercise recommendations for adults with hypertension, emphasizing aerobic exercise as an effective strategy for lowering blood pressure and improving cardiovascular health [2]. In more recent years, the combination of aerobic and resistance exercises has become a key comprehensive strategy to improve functional capacity, cardiovascular health, and overall quality of life in older adults [3], given the age‐related decline in physical independence [4]. Current guidelines recommend 75 or 150 min/week of vigorous or moderate‐intensity activities, including both aerobic and resistance exercises [5, 6]. However, these recommendations provide limited guidance regarding the weekly frequency of exercise sessions that should be performed. The available evidence emphasizes that the effects of exercise training may depend not only on total volume and intensity but also on how this volume is distributed across the week [7]. It has been suggested that patients with hypertension may benefit from daily exercise since the chronic reduction in blood pressure due to regular exercise seems to result from the sum of the acute decreases that follow each exercise bout (post‐exercise hypotension) [8]. Consequently, distributing the same total weekly training volume across more sessions might enhance blood pressure control. However, evidence directly comparing different weekly frequencies of combined training while maintaining equivalent total volume and adhering to current guideline recommendations remains limited, representing a relevant gap in the literature.

Additionally, the implementation of combined aerobic and resistance exercise programs often faces challenges due to barriers such as limited access to appropriate spaces, equipment, and resources [9]. This highlights the need for pragmatic, low‐cost, and easily accessible strategies that promote physical activity through simple, easy‐to‐follow routines that can be reproduced in various settings, without the need for complex instructions or specialized environments. However, exercise training conducted in such a setting has not been evaluated in research studies.

Therefore, this study aimed to assess the effects of a combined exercise program performed in the pragmatic settings either two or four times per week on office and ambulatory blood pressure in adults with hypertension.

## 2 Methods

### 2.1 Study Design and Settings

This randomized, parallel‐group, examiner‐blinded superiority‐controlled trial was performed in Brazil from September 2022 to December 2024. Participants were randomly assigned, using concealed allocation, to one of two 12‐week intervention groups: a combined resistance and aerobic training program performed twice per week (CT2) or four times per week (CT4). To increase the ease of implementation and the generalizability of our findings, exercise training consisted of bodyweight resistance exercises and walking. These exercises were performed on an outdoor athletic track that is publicly available. Ethical approval was obtained from the Hospital de Clinicas de Porto Alegre Ethics Committee, and the trial was registered at ClinicalTrials.gov (NCT04218903), in accordance with the Declaration of Helsinki. The protocol has been previously published in detail elsewhere [10]. This study followed the Consolidated Standards of Reporting Trials (CONSORT) guidelines [11]. All participants provided written informed consent.

### 2.2 Participants

Men and women with a previous diagnosis of hypertension were eligible if they were 50–80 years old, were using up to three classes of antihypertensive medications, and had not engaged in structured exercise programs in the previous three months. Among eligible participants, those who present physical or muscular injuries that limit the performance of the proposed training protocols, have a history of cardiovascular events such as acute myocardial infarction, angina, or stroke within the past 24 months, or present with heart failure classified as New York Heart Association class III or IV are excluded. Additional exclusion criteria included chronic diseases such as cancer, dialysis‐dependent kidney disease, multiple sclerosis, or Parkinson's disease, a body mass index ≥ 40 kg/m^2^, and diabetes mellitus with target organ damage [10]. The participants were recruited from electronic medical records, telephone contacts, social media e‐flyers, word of mouth, and personal referrals. Volunteers first completed a telephone screening, and those who met the preliminary eligibility criteria were subsequently invited for laboratory screening. Participants were instructed to maintain their usual antihypertensive medication, dietary habits and to avoid engaging in any structured exercise or vigorous physical activity on non‐training days throughout the intervention period.

### 2.3 Procedures

The baseline assessment included three morning visits (8:00–11:00 a.m.) to the laboratory at Hospital de Clínicas de Porto Alegre. During the first visit, participants performed office blood pressure measurements, a standardized interview and a comprehensive clinical examination, which included clinical history, anthropometric assessment, resting electrocardiogram, and sit‐stand test. During the second visit, participants repeated blood pressure assessments, completed the World Health Organization Quality of Life (WHOQoL) [12] questionnaire, and were fitted with ambulatory blood pressure monitoring (ABPM). On the following day, participants removed the ABPM devices, underwent handgrip strength testing, and completed a cardiorespiratory fitness assessment. All tests were conducted by two independent evaluators who were blinded to the study interventions. The same protocols were repeated within one week after completion of the 12‐week intervention period, under the same conditions as the baseline assessments.

### 2.4 Randomization and Allocation

The randomization list was generated by an epidemiologist using a web‐based software (www.random.org) with 1:1 allocation and random block sizes that were not disclosed to ensure allocation concealment. Stratified randomization was performed based on sex (male or female), age (50–64 or 65–80 years), and 24‐h systolic blood pressure to ensure balance of these prognostic factors. The epidemiologist did not participate in the recruitment or assignment to intervention groups. The intervention team was blinded to the assessment results, and outcome assessors remained blinded to group allocation throughout the study to minimize potential bias. The participants and the researchers responsible for the training could not be blinded to the experimental groups due to the nature of the interventions.

### 2.5 Outcome Measurements

The primary outcome was the change in 24‐h ambulatory systolic blood pressure from baseline to the 12‐week follow‐up. ABPM was performed over 24 h, using a validated oscillometric device (ABP 2400, Mortara, Milwaukee, USA) with an appropriately sized cuff. The device was programmed to record blood pressure every 15 min during the daytime and every 20 min during the nighttime [13]. Daytime and nighttime periods were primarily defined based on participants' self‐reported wake and sleep times, recorded in a diary, and cross‐validated with the ABPM device records. When unavailable, default times of 7:00 AM (daytime start) and 11:00 PM (nighttime start) were applied. Participants received a diary to record daily activities, symptoms, sleep and wake‐up times, and were instructed to refrain from strenuous physical exercise and alcohol consumption for 24 h before the measurement. Each exam was considered valid when at least 14 daytime and seven nighttime measurements were successfully recorded [13]. In cases of invalid or insufficient recordings, a repeat ABPM assessment was scheduled within 48 h.

Office blood pressure and heart rate were measured using a validated automatic oscillometric device (HBP−1100 monitor; OMRON Healthcare, Kyoto, Japan) after 20 min of rest, with the participant sitting quietly in a chair. Participants were instructed to avoid physical exercise and alcohol ingestion 24 h before the measurement. Three consecutive measurements were taken at 1–2 min apart, on the arm with the highest initial systolic blood pressure. The mean of the last two measurements was used for the analysis of the office blood pressure. Other secondary outcomes included cardiorespiratory fitness, chair‐stand test, isometric handgrip strength, and quality of life. A more detailed description of the assessment procedure is provided in the study protocol paper [10].

### 2.6 Combined Exercise Training Intervention

The combined exercise training program was conducted on an outdoor athletic track. The exercise sessions were conducted in the morning or late afternoon, depending on the participant's preference. The CT2 and CT4 groups performed the same total training volume and overload per week (i.e., minutes of exercise per week, number of sets, number of repetitions, resistance exercises, intervals between sets and repetitions, aerobic exercise modality, and relative intensity of resistance and aerobic training). The only difference between the groups was the number of training sessions per week (two versus four sessions per week).

The training protocol lasted 12 weeks, and participants performed both resistance and aerobic training in sequence in the same session, starting with the bodyweight‐based resistance exercises immediately followed by the aerobic exercise. The training protocol incorporated a planned progression in training volume and was structured into two mesocycles (mesocycle 1: weeks 1–6 and mesocycle 2: weeks 7–12). The complete protocol, including periodization, exercises, volume, and intensity of the combined training, is presented in Table 1.

**TABLE 1:** Combined resistance and aerobic training programs performed twice a week (CT2) and four times a week (CT4).

	CT2		CT4			
	Mesocycle 1	Mesocycle 2	Mesocycle 1		Mesocycle 2	
	Weeks 1–6 (days 1 and 3)	Weeks 7–12 (days 1 and 3)	Weeks 1–6 (days 1 and 3)	Weeks 1–6 (days 2 and 4)	Weeks 7–12 (days 1 and 3)	Weeks 7–12 (days 2 and 4)
**Resistance exercises** (sets × repetitions)						
Push‐up	2 × 10–12	3 × 10–12	2 × 10–12	—	3 × 10–12	—
Squat	3 × 12–15	4 × 12–15	3 × 12–15	—	4 × 12–15	—
Unilateral balance	1 × 30″	1 × 45″	1 × 30″	—	1 × 45″	—
Inverted row	2 × 10–12	3 × 10–12	—	2 × 10–12	—	3 × 10–12
Calf raise	2 × 12–15	2 × 18–20	—	2 × 12–15	—	2 × 18–20
Crunch	2 × 15–20	3 × 20	—	2 × 15–20	—	3 × 20
Intensity, RPE	4–5	5–6	4–5	4–5	5–6	5–6
**Walking/Running**						
Volume, min	40	50	20	20	25	25
Intensity, RPE	5–6	5–6	5–6	5–6	5–6	5–6

All training sessions were conducted under the supervision of researchers and experienced supervisors. The intensity of the training sessions was monitored using the Borg's modified CR10 rating of perceived exertion (RPE) scale [14]. For the bodyweight‐based resistance exercises, the target exercise intensity was 4–6 RPE [15]. Aerobic exercise was performed at 5–6 RPE [16]. The rate of perceived exertion was monitored during the first session of each week to guide intensity adjustments. In resistance training, it was assessed 8–10s into the first set of each exercise to evaluate and rate their muscular sensations. In the aerobic training, it was recorded every 400 m lap to adjust pace based on the overall perception of muscle fatigue, shortness of breath, and physical stress. These adjustments ensured that participants in both groups trained at the same target RPE throughout the study. The internal training load was estimated using an additional RPE assessed 15 min after the completion of exercise in the first and the last weeks of each mesocycle (session RPE × duration) [17].

Heart rate (HR) was monitored using a chest monitor (Polar H10, Finland) continuously at weeks 1–2 of the first mesocycle. These measures aimed to help understand potential differences between the target intensity of a prescription via RPE and the actual physiological intensity of the exercise session via HR. The individual maximal HR (HR_max_) was determined according to the highest HR value achieved during the cardiorespiratory test. For HR at rest (HRrest), the lowest value recorded during visits 1 and 2 of the baseline period was considered. To determine the relative exercise intensity, the percentage of HR reserve (HR_reserve_) utilized during each session was calculated using the formula: [(HRexercise − HRrest) / (HRmax − HRrest)] × 100 [18].

The bodyweight resistance exercises included push–ups performed on street workout bars, inverted rows using an overhead strap attached to a bar, calf raises on a step, squats, and abdominal exercises on floor mats. Adjustments to these exercises were made as needed. Common modifications included switching from bilateral to unilateral movements for lower limb exercises and changing joint angles during push‐ups and inverted rows. Further details on the exercises, progression methods, and variations are found in the Supporting Information (File S1).

### 2.7 Sample Size and Power Calculation

A sample size of 98 individuals with hypertension was calculated based on a 1:1 allocation ratio (CT2, *n* = 49; CT4, *n* = 49), allowing for a 10% anticipated dropout rate. The calculation aimed to detect a between‐group difference of 4 ± 10 mmHg in 24‐h systolic blood pressure, with 80% statistical power and a two‐sided type I error rate of 5%. This value was determined based on a meta‐analysis of combined training interventions with the same population [19] and represents a magnitude considered clinically meaningful for cardiovascular risk reduction [20]. The sample size was estimated using the WinPepi software calculator.

### 2.8 Statistical Analyses

The endpoints were analyzed using a full analysis set that included all randomized participants, following the intention‐to‐treat principle. A secondary per‐protocol analysis was performed including participants who maintained stable antihypertensive medication throughout the study and achieved at least 80% adherence to the assigned (≥ 19 sessions for participants allocated to CT2 and ≥ 38 sessions for CT4). Data tabulation was performed independently in duplicate by two researchers, and discrepancies were resolved through comparison to ensure data accuracy. The statistician did not participate in recruitment or assignment to the experimental sessions and was blinded to the interventions.

The normality assumption was assessed using the Shapiro–Wilk test and visual inspection of Q–Q plots. The continuous variables were analyzed using the independent samples *t*‐test, whereas the categorical variables were analyzed using the chi‐squared test. Primary and secondary outcomes were analyzed using the Generalized Estimating Equations, considering the group factor (CT2 and CT4), the time factor (pre‐intervention and post‐training), and the interaction between group and time. The models were adjusted for use of angiotensin‐converting enzyme inhibitors due to baseline differences observed before the intervention, as well as for other variables that may influence blood pressure outcomes, including baseline values, body mass index, age, and sex. Post hoc comparisons were run using the sequential Bonferroni test to adjust for multiple testing. Effect sizes were calculated using Cohen's d, and the interpretation of the effect size adopted was based on the following criteria: less than 0.50, small; 0.50–0.79, medium; and at least 0.80, large. The inter‐individual blood pressure variability was calculated using the delta values (post‐ minus pre‐blood pressure). To categorize the participants as Responders, we designated the clinically meaningful change of reduction in systolic BP (4 mmHg) or diastolic BP (2 mmHg). These cut‐off values were derived from the studies used to inform the sample size calculation [19, 20]. Statistical significance was accepted at P less than 0.05, and a trend toward significance was detected for P values ranging from 0.05 to 0.10. All analyses were performed using SPSS Statistics for Windows (version 22.0; IBM, Armonk, New York, USA).

## 3 Results

**FIGURE 1:**
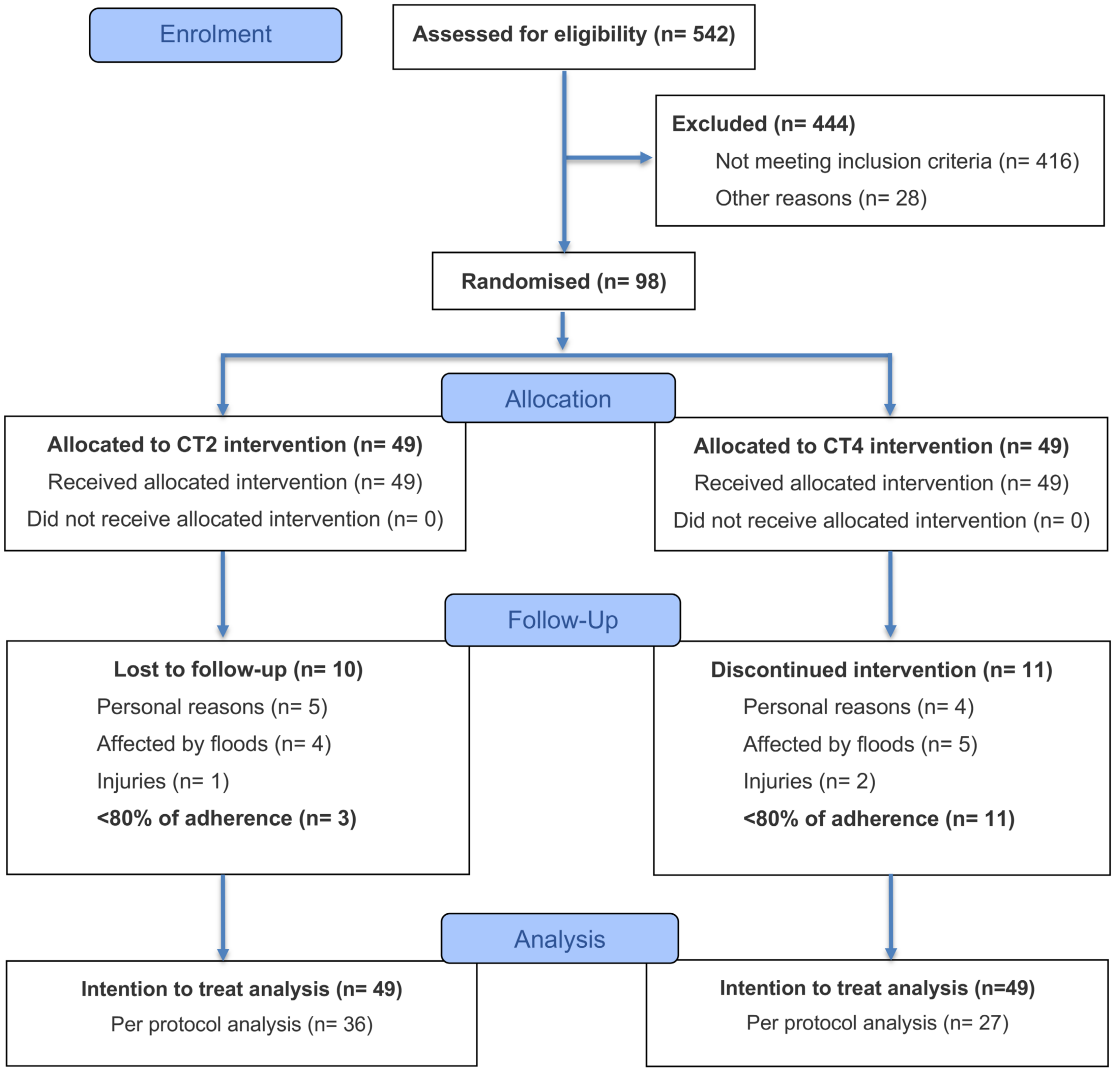
Flow diagram of participants in the interventions involving combined resistance and aerobic training program performed twice (CT2) or four times per week (CT4).

Among 542 individuals screened for eligibility, 98 met the inclusion criteria and were randomly assigned to either the CT2 group (*n* = 49) or the CT4 group (*n* = 49) (Figure 1). Twenty‐one participants did not complete the post‐intervention assessments (CT4 *n* = 11; CT2 *n* = 10). Reasons for dropout include the severe floods that occurred in Porto Alegre, Brazil (*n* = 9) (one of the worst natural disasters in the region's history) as well as lack of time (*n* = 3) and family‐related issues (*n* = 6). Adverse events were limited to femoral fractures incurred from a fall at home (one participant in CT2), ankle sprain sustained during a commute to work, a pre‐existing cervical spine issue (two participants in CT4), and were judged to be independent of the training sessions. Fourteen participants (CT4 *n* = 11; CT2 *n* = 3) failed to complete at least 80% of the prescribed sessions. A total of 63 participants were included in the per‐protocol analysis.

Baseline characteristics including all randomized participants are presented in Table 2. The sample consisted of older adults, predominantly white, who were overweight or obese. Baseline values were well‐balanced between groups, with no significant differences observed except for the use of ACE inhibitors. Supporting Information (File S2) summarizes the baseline characteristics of the participants who completed the study protocol (per‐protocol analysis) and demonstrated similar values between groups at baseline.

**TABLE 2:** Baseline demographic and clinical characteristics of the participants who performed the combined exercise training twice a week (CT2) and four times a week (CT4).

Variables	CT2 (*n* = 49)	CT4 (*n* = 49)	*P*
Men/women, n	20/29	20/29	1.000
Age, years	63 ± 7	62 ± 7	0.893
Body weight, kg	78 ± 2	79 ± 2	0.881
Height, cm	165 ± 8	164 ± 9	0.872
BMI, kg/m^2^	29 ± 1	29 ± 1	0.578
Waist, cm	98 ± 2	99 ± 2	0.733
**Ethnicity, n(%)**			
White	27 (75)	19 (70)	0.083
Black	3 (8)	7 (26)	
Indigenous	6 (17)	1 (4)	
**Anti‐hypertensive medications, n(%)**			
Diuretics	28 (57)	23 (47)	0.419
β‐Blockers	14 (29)	10 (20)	0.482
ARBs	26 (53)	22 (45)	0.545
CCB	18 (37)	9 (18)	0.069
ACEI	10 (20)	23 (47)*	**0.005**
Combined therapy	31 (63)	31 (63)	1.000
**Comorbidities, n(%)**			
Diabetes mellitus	10 (20)	6 (12)	0.413
Hypercholesterolemia	27 (55)	21 (43)	0.312

*Note:* Values are mean ± SD. Bold *p*‐values indicate significant results (*P* < 0.05).

Abbreviations: ACEI, Angiotensin converting enzyme inhibitors; ARBs, Angiotensin II receptor blockers; CCB, Calcium channel blockers.

* CT2 different from CT4 (*p* < 0.05).

### 3.1 Combined Exercise Training Sessions

In the intention‐to‐treat analysis, attendance rates were 75% ± 5% in CT2 (18 sessions) and 62% ± 5% in CT4 (31 sessions), showing a trend toward significance between groups (*p* = 0.083). In the per‐protocol analysis, attendance was significantly higher in the CT2 (92% ± 9%; 23 sessions) than in the CT4 (85% ± 16%; 41 sessions) (*p* = 0.02). The sessions missed were primarily due to adverse weather conditions and personal or family obligations. No training‐related adverse events were reported. Minor musculoskeletal discomfort, consistent with delayed onset muscle soreness, occurred in a few participants during the first two weeks of familiarization. These were expected physiological responses that required no medical intervention or training interruption.

Initial exercise intensity (weeks 1–2) was comparable between groups, reflecting a standardized physiological demand at baseline for both groups (CT4: 63% ± 5% vs. CT2: 60% ± 8% HR_reserve_; *p* = 0.79). Internal training load (Session‐RPE × session duration × weekly frequency) progressively increased in both groups throughout the intervention. However, CT2 consistently exhibited a higher internal load than CT4 at weeks 1–2 (CT2: 332 ± 25 AU vs. CT4: 272 ± 17 AU; *p* = 0.048), week 6 (CT2: 347 ± 26 AU vs. CT4: 276 ± 13 AU; *p* = 0.012), week 8 (CT2: 452 ± 38 AU vs. CT4: 337 ± 27 AU; *p* = 0.012), and week 12 (CT2: 430 ± 24 AU vs. CT4: 316 ± 35 AU; *p* = 0.008).

### 3.2 Ambulatory Blood Pressure

**FIGURE 2:**
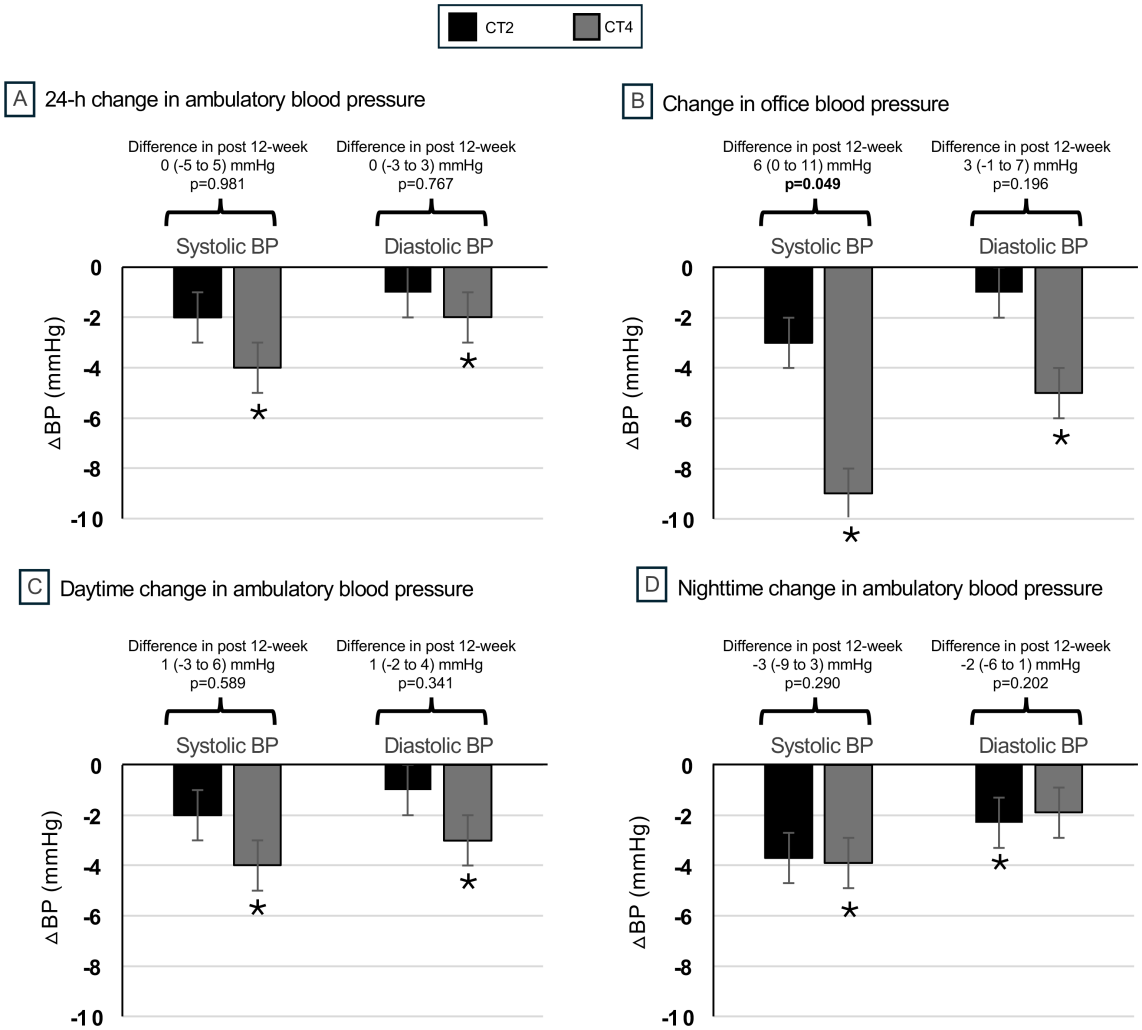
Changes in Office and Ambulatory Blood Pressure after 12 Weeks of Combined Exercise Training for participants who completed the protocol with an attendance rate of at least 80%. Intervention groups: Combined resistance and aerobic training program performed twice per week (CT2) or four times per week (CT4). Data are presented as mean ± standard error; *indicate significant difference in values between the post‐training vs. baseline.

The baseline 24‐h ambulatory blood pressure values were similar between groups. The intention‐to‐treat analysis showed no significant differences between the groups for any ambulatory blood pressure variables. In the per‐protocol analysis, the CT4 group showed significant reductions in 24‐h systolic (*p* = 0.012) and diastolic (*p* = 0.010) blood pressure after 12 weeks of training. Additionally, reductions were also observed in the CT4 group for daytime systolic (*p* = 0.015) and diastolic (*p* = 0.009) blood pressure, as well as nighttime systolic blood pressure (*p* = 0.041). In the CT2 group, a significant reduction was found in daytime diastolic blood pressure after training (*p* = 0.022) (Table 3 and Figure 2).

**TABLE 3:** Ambulatory blood pressure at baseline and after the 12‐week intervention programs using combined resistance and aerobic training program performed twice per week (CT2) or four times per week (CT4).

Intention‐to‐treat analyses	CT2 (*n* = 49)					CT4 (*n* = 49)					Between‐Group difference in final	*P*	Effect size
	Baseline	Final	∆	*P*	Effect size	Baseline	Final	∆	*P*	Effect size			
**24 h**													
SBP	124 ± 2	123 ± 2	−1 (−5 to 2)	0.377	0.1	124 ± 2	123 ± 1	−1 (−4 to 3)	0.790	0.1	0 (−5 to 4)	0.824	0.0
DBP	76 ± 1	75 ± 1	−1 (−2 to 1)	0.456	0.1	77 ± 1	75 ± 1	−2 (−3 to 0)	0.071	0.3	0 (−3 to 3)	0.946	0.0
HR	71 ± 2	70 ± 1	−1 (−3 to 2)	0.481	0.1	69 ± 1	69 ± 1	0 (−2 to 2)	0.746	0.0	1 (−3 to 6)	0.442	0.1
**Daytime**													
SBP	128 ± 2	127 ± 2	‐1 (−5 to 2)	0.459	0.1	127 ± 2	126 ± 2	‐1 (−4 to 3)	0.733	0.1	1 (−4 to 5)	0.765	0.1
DBP	79 ± 1	78 ± 1	‐1 (−2 to 1)	0.523	0.1	79 ± 1	77 ± 1	−2 (−4 to 0)	0.093	0.3	1 (−2 to 4)	0.565	0.1
HR	74 ± 2	73 ± 2	‐1 (−3 to 2)	0.564	0.1	71 ± 1	71 ± 2	0 (−2 to 3)	0.966	0.0	‐2 (−3 to 6)	0.471	0.1
**Nighttime**													
SBP	114 ± 2	111 ± 2	−3 (−7 to 1)	0.187	0.2	115 ± 2	115 ± 2	0 (−4 to 4)	0.944	0.0	−4 (−10 to 2)	0.172	0.3
DBP	69 ± 1	67 ± 1	−2 (−3 to 0)	0.084	0.3	70 ± 1	69 ± 1	−1 (−3 to 1)	0.511	0.1	−2 (−5 to 1)	0.180	0.3
HR	65 ± 2	63 ± 2	−2 (−4 to 1)	0.223	0.1	63 ± 1	62 ± 1	−1 (−3 to 1)	0.290	0.1	1 (−5 to 5)	0.540	0.1

Per protocol analyses	CT2 (*n* = 36)					CT4 (*n* = 27)					Between‐Group difference in final	*P*	Effect size
	Baseline	Final	∆	*P*	Effect size	Baseline	Final	∆	*P*	Effect size			
**24 h**													
SBP	125 ± 2	123 ± 2	−2 (−6 to 1)	0.160	0.2	126 ± 2	122 ± 1	−4 (−7 to −1)	**0.012**	0.5	0 (5 to 5)	0.981	0.2
DBP	77 ± 1	76 ± 1	−1 (−3 to 1)	0.148	0.2	77 ± 1	75 ± 1	−2 (−4 to −1)	**0.010**	0.4	0 (−3 to 3)	0.767	0.2
HR	69 ± 2	68 ± 2	−1 (−4 to 1)	0.408	0.1	70 ± 2	69 ± 2	−1 (−3 to 2)	0.636	0.1	−2 (−6 to 3)	0.451	0.1
**Daytime**													
SBP	129 ± 2	127 ± 2	−2 (−6 to 1)	0.231	0.2	130 ± 2	126 ± 1	−4 (−7 to −1)	**0.015**	0.5	1 (−3 to 6)	0.589	0.1
DBP	80 ± 1	79 ± 1	−1 (−3 to 1)	0.218	0.2	80 ± 1	77 ± 1	−3 (−5 to −1)	**0.009**	0.6	1 (−2 to 4)	0.341	0.4
HR	71 ± 2	70 ± 2	−1 (−3 to 2)	0.451	0.1	72 ± 2	72 ± 2	0 (−3 to 3)	0.891	0.0	−2 (−7 to 3)	0.440	0.2
**Nighttime**													
SBP	115 ± 3	111 ± 2	−4 (−8 to 1)	0.100	0.3	118 ± 3	114 ± 2	−4 (−7 to 0)	**0.045**	0.3	−3 (−9 to −3)	0.290	0.3
DBP	69 ± 1	67 ± 1	−2 (−4 to −1)	**0.022**	0.3	71 ± 1	69 ± 1	−2 (−4 to 0)	0.103	0.4	−2 (−6 to −1)	0.202	0.4
HR	63 ± 2	61 ± 2	−2 (−5 to 1)	0.200	0.2	64 ± 2	63 ± 1	−1 (−3 to 1)	0.200	0.1	−2 (−6 to 2)	0.328	0.2

*Note:* Values are mean ± SD or mean (95% CI). Bold *p*‐values indicate significant results (*p* < 0.05). Intervention groups: combined resistance and aerobic training program performed twice per week (CT2) or four times per week (CT4).

Abbreviations: DBP, diastolic blood pressure (mmHg); HR: heart rate (bpm); SBP: systolic blood pressure (mmHg).

### 3.3 Office Blood Pressure

Office blood pressure values were similar between groups at baseline. The intention‐to‐treat analysis following a 12‐week intervention revealed significant reductions in the CT4 group for systolic blood pressure (*p* < 0.001) and diastolic blood pressure (*p* = 0.003). In the CT2 group, significant reductions were observed in systolic blood pressure (*p* = 0.032) compared with baseline. In the per‐protocol analysis, the CT4 group exhibited significantly lower systolic blood pressure compared with CT2 (*p* = 0.049) after 12 weeks. In addition, within the CT4 group, significant decreases were observed in systolic and diastolic blood pressure (*p* < 0.001) compared with the baseline. No significant changes in these variables were found within the CT2 group (Table 4 and Figure 2).

**TABLE 4:** Office blood pressure at baseline and after the 12‐week intervention programs of combined resistance and aerobic training program performed twice per week (CT2) or four times per week (CT4).

Intention‐to‐treat analyses	CT2 (*n* = 49)					CT4 (*n* = 49)					Between‐Group difference in final	*P*	Effec t size
	Baseline	Final	∆	*P*	Effect size	Baseline	Final	∆	*P*	Effect size			
SBP	132 ± 2	128 ± 2	−4 (−8 to 0)	0.032*	0.3	132 ± 2	124 ± 2	−8 (−10 to −5)	< 0.001*	0.6	3 (−2 to 8)	0.174	0.3
DBP	77 ± 1	75 ± 1	−2 (−4 to 0)	0.116	0.3	77 ± 1	74 ± 1	−3 (−6 to −1)	0.003*	0.4	1 (−2 to 5)	0.471	0.1
HR	72 ± 2	70 ± 2	−2 (−4 to 1)	0.187	0.1	71 ± 1	70 ± 1	−1 (−4 to 1)	0.330	0.1	0 (−5 to 5)	0.872	0.0

Per protocol analyses	CT2 (*n* = 36)					CT4 (*n* = 27)					Between‐Group difference in final	*P*	Effect size
	Baseline	Final	∆	*P*	Effect size	Baseline	Final	∆	*P*	Effect size			
SBP	133 ± 2	130 ± 2	−3 (−7 to 1)	0.093	0.3	133 ± 2	124 ± 2	−9 (−12 to 6)	< 0.001*	0.8	6 (0 to 11)	0.049*	0.5
DBP	77 ± 1	76 ± 1	−1 (−3 to 1)	0.399	0.2	78 ± 1	73 ± 1	−5 (−7 to −3)	< 0.001*	0.8	3 (−1 to 7)	0.196	0.5
HR	69 ± 2	67 ± 2	−2 (−5 to 1)	0.146	0.2	72 ± 2	71 ± 2	−1 (−4 to 2)	0.446	0.1	−4 (−9 to 2)	0.212	0.4

*Note:* Values are mean ± SD or mean (95% CI). *indicate significant results (*p* < 0.05). Intervention groups: combined resistance and aerobic training program performed twice per week (CT2) or four times per week (CT4).

Abbreviations: DBP, diastolic blood pressure (mmHg); HR, heart rate (bpm); MAP, mean arterial pressure (mmHg); SBP, systolic blood pressure (mmHg).

### 3.4 Responders for Blood Pressure

**FIGURE 3:**
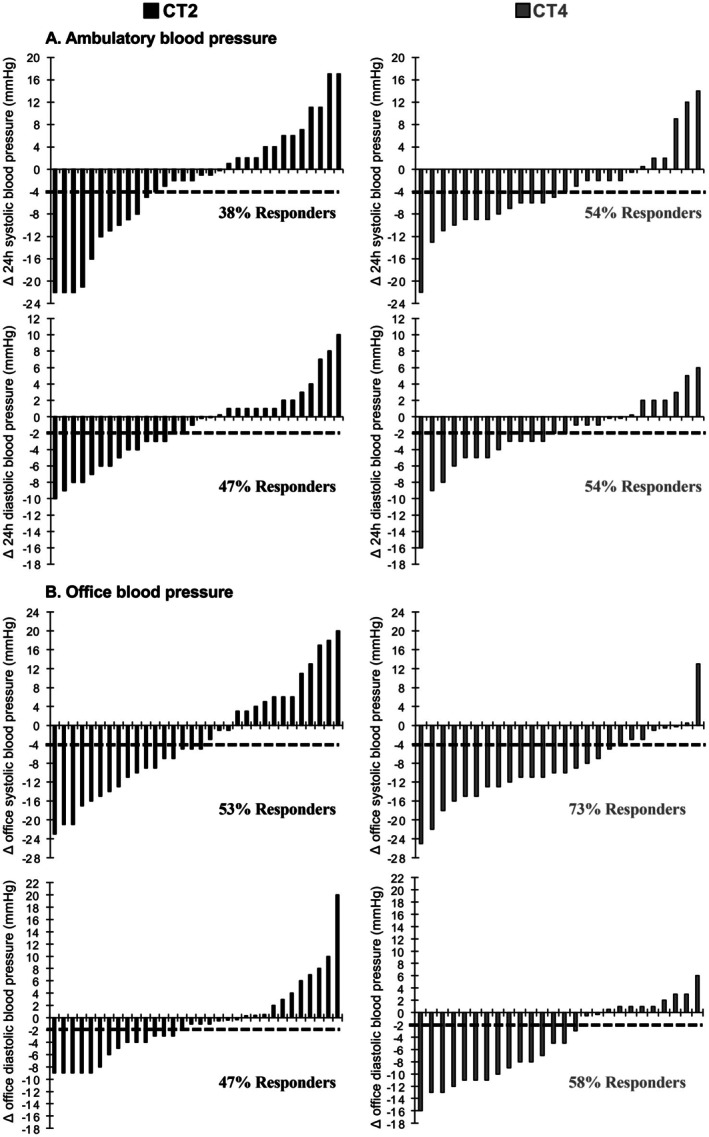
Individual changes in 24‐h ambulatory (A) and office (B) systolic and diastolic blood pressure (final minus baseline) who completed the protocol with an attendance rate of at least 80%. Intervention groups: Combined resistance and aerobic training program performed twice per week (CT2) or four times per week (CT4). Dashed line: Minimal detectable change for systolic (4 mmHg) and diastolic blood pressure (2 mmHg).

The proportions of responders in both groups are shown in Figure 3. For 24‐h blood pressure, 38% of participants in CT2 and 54% in CT4 were classified as responders for systolic, and 47% in CT2 and 54% in CT4 for diastolic blood pressure. For office blood pressure, responder proportions were 53% (CT2) and 73% (CT4) for systolic, and 47% (CT2) and 58% (CT4) for diastolic blood pressure.

### 3.5 Physical Fitness and Quality of Life

The results for physical fitness and quality of life were consistent across intention‐to‐treat and per‐protocol analyses. Significant improvements in cardiorespiratory fitness after the training period were observed in the CT4 group (*p* < 0.05), whereas no significant changes were detected in CT2. In the chair‐stand test, both groups improved the number of repetitions and the five‐rep completion time (*p* < 0.001) after training. The handgrip strength did not improve in both groups after the 12‐week exercise program. In the quality‐of‐life questionnaire, the psychological domain improved in CT2 (*p* = 0.020) and showed tendency in CT4 (*p* = 0.051) at post‐training. No group differences were observed in any physical fitness and quality of life measurements (Table 5).

**TABLE 5:** Physical fitness and quality of life measurements at baseline and after the 12‐week intervention programs using combined resistance and aerobic training program performed twice per week (CT2) or four times per week (CT4).

Intention‐to‐treat analyses	CT2 (*n* = 49)					CT4 (*n* = 49)					Between‐Group difference in final	*P*	Effect size
	Baseline	Final	∆	*P*	Effect size	Baseline	Final	∆	*P*	Effect size			
**Cardiorespiratory fitness**													
Peak VO2	27 ± 1	28 ± 1	1 (−1 to 2)	0.407	0.1	25 ± 1	27 ± 1	2 (1 to 3)	0.001*	0.3	1 (−2 to 3)	0.676	0.1
Distance (m)	1080 ± 38	1106 ± 49	26 (−43 to 95)	0.460	0.1	1045 ± 35	1143 ± 42	97 (37 to 158)	0.002*	0.4	−37 (−165 to 91)	0.571	0.1
**Chair‐stand test**													
Repetitions	19 ± 1	24 ± 1	5 (4 to 6)	< 0.001*	0.7	19 ± 1	23 ± 1	4 (3 to 5)	< 0.001*	0.6	1 (−1 to 3)	0.234	0.1
Five‐time (s)	8 ± 1	7 ± 1	−1 (−1 to 0)	< 0.001*	0.1	9 ± 1	7 ± 1	−2 (−1 to 0)	< 0.001*	0.3	−0 (−1 to 1)	0.470	0.0
**Handgrip test (kg)**													
Dominant arm	32 ± 1	35 ± 2	3 (1 to 5)	0.012*	0.3	34 ± 2	34 ± 2	0 (−2 to 2)	0.934	0.0	1 (−3 to 6)	0.561	0.1
Non‐dominant arm	32 ± 1	34 ± 2	2 (0 to 5)	0.097	0.2	32 ± 1	34 ± 2	2 (0 to 5)	0.164	0.2	0 (−4 to 5)	0.909	0.0
**Quality of life (points)**													
Physical	69 ± 2	69 ± 2	0 (−4 to 4)	0.946	0.0	63 ± 2	65 ± 2	2 (−2 to 6)	0.325	0.1	4 (−2 to 10)	0.214	0.3
Psychological	68 ± 2	72 ± 2	4 (1 to 7)	0.013*	0.3	65 ± 2	70 ± 2	5 (1 to 8)	0.010*	0.4	2 (−3 to 7)	0.479	0.1
Social relationships	69 ± 2	70 ± 2	1 (−3 to 5)	0.521	0.1	68 ± 2	68 ± 2	0 (−4 to 4)	0.971	0.0	2 (−4 to 8)	0.457	0.1
Environment	67 ± 2	67 ± 2	0 (−3 to 2)	0.723	0.0	67 ± 2	68 ± 2	1 (−3 to 5)	0.555	0.1	−1 (−7 to 4)	0.632	0.1

*Note:* Values are mean ± SD or mean (95% CI). VO2: volume of oxygen (mL.kg^−1^.min^−1^); *p values indicate significant results (*p* < 0.05). Intervention groups: combined resistance and aerobic training program performed twice per week (CT2) or four times per week (CT4).

Abbreviation: VO2, volume of oxygen (mL.kg^−1^.min^−1^).

## 4 Discussion

To our knowledge, this is the first randomized controlled trial to directly evaluate the influence of different exercise frequencies on both office and ambulatory blood pressure in older adults with essential hypertension. Although no significant between‐group differences were observed for ambulatory blood pressure, the exploratory per‐protocol results showed reductions in office blood pressure favoring the higher frequency group. Additionally, within‐group improvements after training and a higher proportion of responders for 24 h blood pressure in CT4 suggest that training frequency may act as a modulator of blood pressure responsiveness, but further powered trials are needed to confirm these observations.

The observed magnitude of reduction in 24‐h systolic blood pressure (~4 mmHg) in the CT4 group aligns with what is epidemiologically recognized as a clinically meaningful change [20] but must be interpreted with caution as it does not substitute for the lack of statistically significant between‐group differences in our primary outcome. This absence of differences between groups in ambulatory blood pressure data may be attributed to lack of power due to the limited sample size of the study. Although the intention‐to‐treat analysis remains the gold standard for clinical effectiveness, the per‐protocol approach provides a more precise test of our primary physiological hypothesis. By including only those who strictly adhered to the sessions, we could better isolate the somatic effect of frequent exercise stimuli on chronic BP adaptations. Our per‐protocol analysis demonstrated significant between‐group differences in office systolic blood pressure, favoring the CT4 protocol. This finding is complemented by a higher rate of responders in the CT4 group compared with CT2, suggesting that distributing the exercise dose across more days may enhance the probability of achieving a meaningful blood pressure reduction. The cumulative effect of more frequent episodes of post‐exercise hypotension could play a role in this potential advantage of CT4 [19, 21, 22, 23, 24]. Other potential mechanisms associated with blood pressure changes such as endothelial function and autonomic function [25] were not measured in this study and remain speculative. These exploratory, hypothesis‐generating findings should be interpreted cautiously and do not replace the primary intention‐to‐treat analysis.

The intervention implemented in this study represents a pragmatic training program, consisting primarily of bodyweight resistance exercises and walking, which is low‐cost, accessible, and feasible for implementation in public spaces or at home. The use of RPE for intensity modulation further enhances its pragmatism, particularly for populations facing environmental and psychosocial barriers to exercise, such as those with hypertension. Although initial relative intensity was similar between groups, CT2 yielded a higher internal load throughout the study, which can be explained by the longer session duration in CT2, as previously reported in the literature [26, 27]. The lower internal load observed in CT4 suggests that distributing training volume into shorter, more frequent sessions may reduce the session RPE. The reduction in perceived strain could represent a strategic approach to lowering psychological barriers to exercise such as post‐exercise fatigue [28], but its direct impact on long‐term adherence in community settings remains to be confirmed.

Aerobic fitness as assessed by maximal oxygen consumption improved in CT4 but not in CT2 after 12 weeks of training, suggesting another advantage of higher weekly frequency of exercise in older adults. This improvement is clinically meaningful, particularly for the sample of older adults with hypertension who exhibit reduced aerobic capacity, where gains of 2–3 mL/kg/min are linked to substantial reductions in cardiovascular risk and mortality [29]. Given that both groups experienced the same weekly training volume and relative intensity, our findings underscore exercise frequency as a critical determinant of these observed gains in the CT4 group.

The improvement of muscular strength represents a primary objective of any exercise intervention in older adults since preserving the functional capacity with advancing age is related to the maintenance of the neuromuscular system [30]. The sit‐to‐stand test is widely employed as a surrogate measure of lower‐limb muscle strength, balance control, and functional capacity in older individuals, as well as a predictor of all‐cause and cardiovascular mortality [31]. Clinically established thresholds for muscle weakness (defined as grip strength < 26 kg in men and < 16 kg in women) have been proposed as reliable biomarkers of age‐related functional decline and increased risk of premature mortality [32]. The implemented exercise program, regardless of weekly frequency, elicited neuromuscular benefits in the participants. These findings demonstrate that this pragmatic physical activity strategy, combining calisthenic exercises with walking/running, confers significant functional benefits in older adults.

Health‐related quality of life is associated with physical fitness in older adults, and improvements in fitness parameters may enhance quality of life [33]. In this study, the psychological domain of quality of life improved after 12 weeks of intervention in both groups. This aligns with previous findings showing that combined interventions, such as 10 weeks of dance classes with resistance training, led to greater improvements in this domain compared with dance alone in older women [34]. An unexpected finding was the lack of improvement in overall quality of life, and particularly in the physical domain. This may be partly explained by the initially high baseline scores in our sample, which were higher than those reported in the aforementioned study despite the use of the same questionnaire [35]. Therefore, the high initial scores could have masked small but clinically relevant improvements in quality of life.

Our study addresses a novel and clinically relevant question, representing a significant advancement in understanding exercise prescription for hypertension. It provides the first randomized clinical trial evidence on how exercise frequency, rather than total volume, impacts blood pressure control in older adults with hypertension. A key strength of our study lies in its design, as both the exercise prescriptions with different frequencies involved the same total weekly training volume. This careful design allowed us to isolate the specific effect of exercise frequency while controlling total training volume as well as exercise intensity, making our findings particularly robust for informing future exercise guidelines. A critical pragmatic aspect of our exercise intervention is its accessibility and real‐world applicability. The implementation of an exercise protocol composed of walking/jogging associated with bodyweight‐exercises underscores the importance of feasibility in public health, facilitating long‐term adherence in community settings. Our protocol was designed to be implemented in casual settings without specialized equipment, relying on simple tools for exercise intensity control. By using RPE for intensity control, participants can self‐regulate their effort, ensuring the intervention is tolerable and adaptable to individual daily variations. Even recognizing that RPE‐based intensity prescription is subject to individual variability in perception among older adults, it remains the most accessible and practical tool for monitoring internal load in this population.

This study has limitations that should be considered when interpreting the results. Our sample consisted of older adults with controlled hypertension, and the results may not be directly extrapolatable to younger populations, normotensive individuals, or patients with severe hypertension and associated clinical complications. The COVID−19 pandemic and an unprecedented flood in the state of Rio Grande do Sul (May 2024) extended the data collection period and hindered the acquisition of some planned outcomes (e.g., flow‐mediated dilation and glycated hemoglobin). These events also affected participant adherence during recruitment phases. The flood severely disrupted local infrastructure and public services, leading to a temporary suspension of data collection and the relocation of assessments to ensure participant safety and operational feasibility. This period coincided with the highest participant dropout rate (*n* = 9). Thus, these unforeseen circumstances resulted in a final sample size smaller than initially planned to detect the predefined margin of superiority. Consequently, the absence of significant differences between the groups should be interpreted with caution, as it may reflect insufficient statistical power. Future studies should aim to confirm these findings and explore the underlying physiological mechanisms contributing to the observed outcomes.

### 4.1 Perspectives

Our findings highlight a clinical trade‐off between exercise adherence and physiological efficacy. Although lower frequency favored adherence, distributing the same weekly volume over more sessions may offer additional benefit among adherent participants. The potential advantage of higher frequency on blood pressure control may be partially explained by the cumulative effect of post‐exercise hypotension, as exercising more days per week represents more time under the acute BP‐lowering effects of each session.

Future research is warranted to validate these hypothesis‐generating findings in larger trials addressing the long‐term effectiveness of volume‐matched protocols in individuals with hypertension. Given that current clinical guidelines primarily emphasize total weekly exercise volume with limited guidance on its distribution, our work advances efforts to understand how varying training frequency within a pragmatic protocol impacts blood pressure control.

## Funding

This work was supported by FIPE/HCPA, grant number: 18‐0642.

## Conflicts of Interest

The authors declare no conflicts of interest.

## Supporting information


**Data S1:** Supporting Information.

## Data Availability

The data that support the findings of this study are available from the corresponding author upon reasonable request.
