# Supplementary material for: Differential Effects of Twice vs. Four Times Weekly Combined Exercise Training in Aging Adults With Hypertension: A Randomized Clinical Trial
Source: Scand J Med Sci Sports. 2026 Mar 6;36(3):e70239. doi: 10.1111/sms.70239 (PMC12966632; doi:10.1111/sms.70239)
Supplement: Supplementary file 1 — Data S1: Supporting Information. [file SMS-36-e70239-s001.docx]

**Supplementary Material**


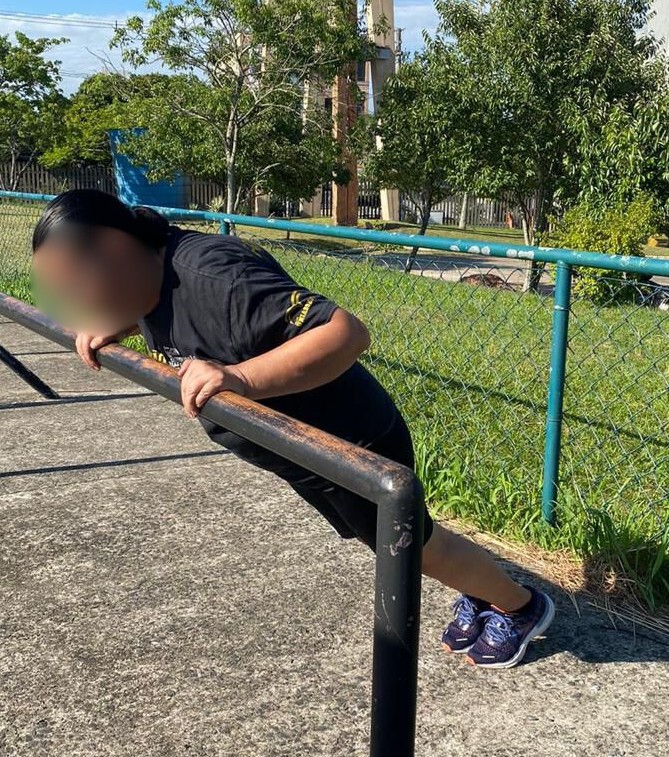

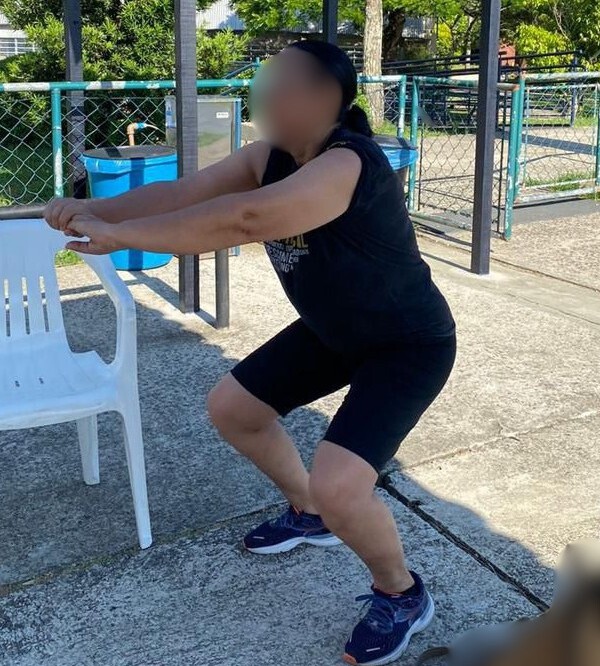

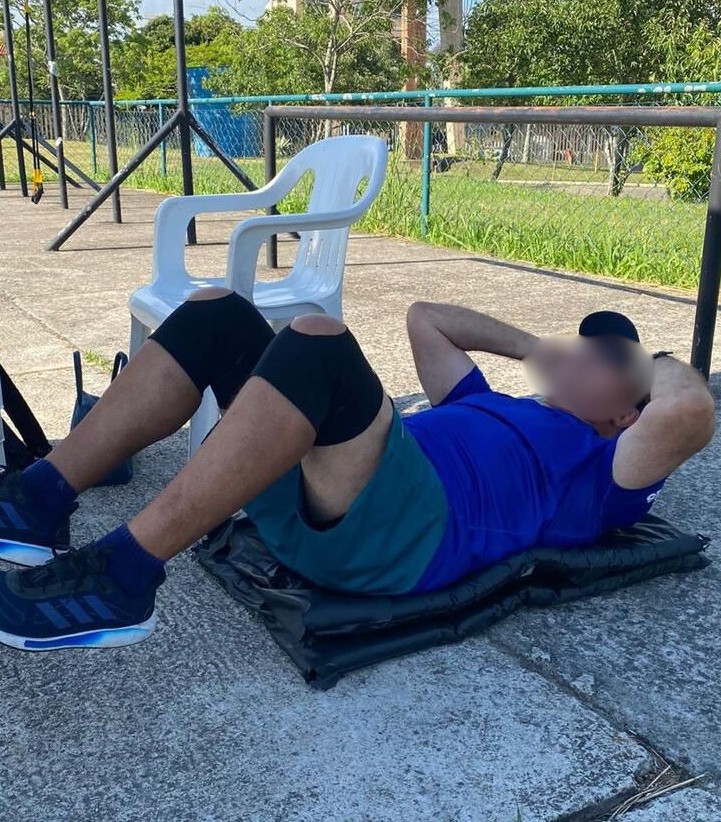

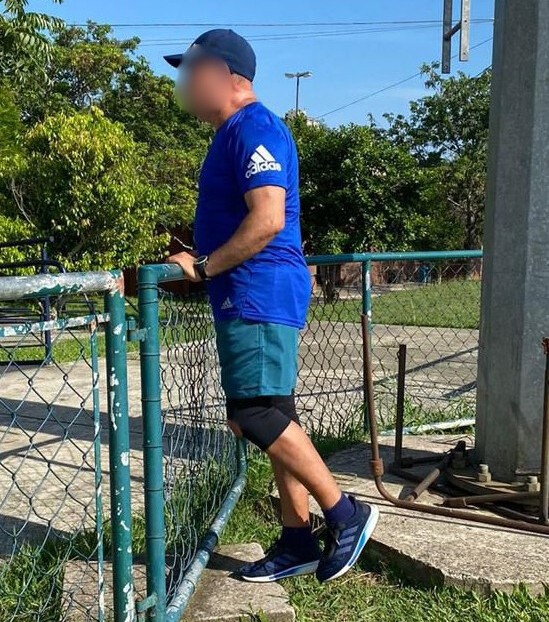

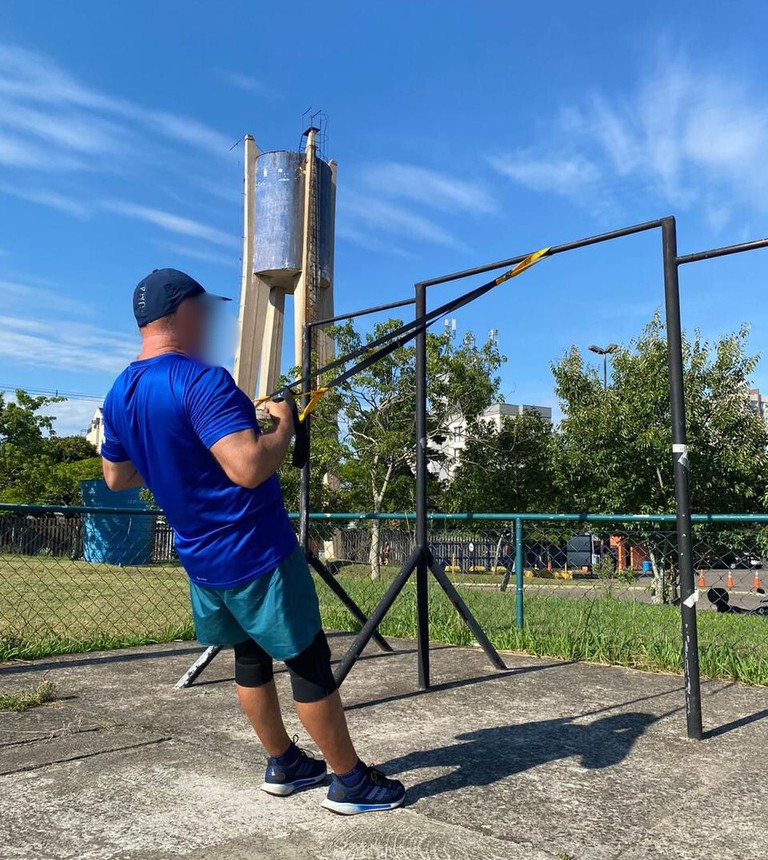
**S1 - Bodyweight Resistance Exercises**

**Inverted Row**

**Setup:** body positioned under the strap attached to a bar, heels on the ground. **Execution:** pulling the torso toward the strap with scapular retraction.

**Intensity adjustment:** body angle adjusted relative to the ground.

**Squat**

**Setup:** standing position, feet shoulder- width apart.

**Execution:** controlled hip and knee flexion to a comfortable depth, followed by extension.

**Intensity adjustment:** depth of squat, pace of movement, or performing the exercise unilaterally.

**Crunch**

**Setup:** supine position on a mat, hips and knees flexed, feet off the ground.

**Execution:** lifting the head and upper trunk off the mat through trunk flexion. **Intensity adjustment:** trunk flexion range of motion and pace movement.

**Calf Raise**

**Setup:** forefoot positioned on a step, heels unsupported, hands lightly holding a support if needed.

**Execution:** raising the body by ankle plantar flexion and lowering the heels. **Intensity adjustment;** range of motion, pace of movement, or performing the exercise unilaterally.


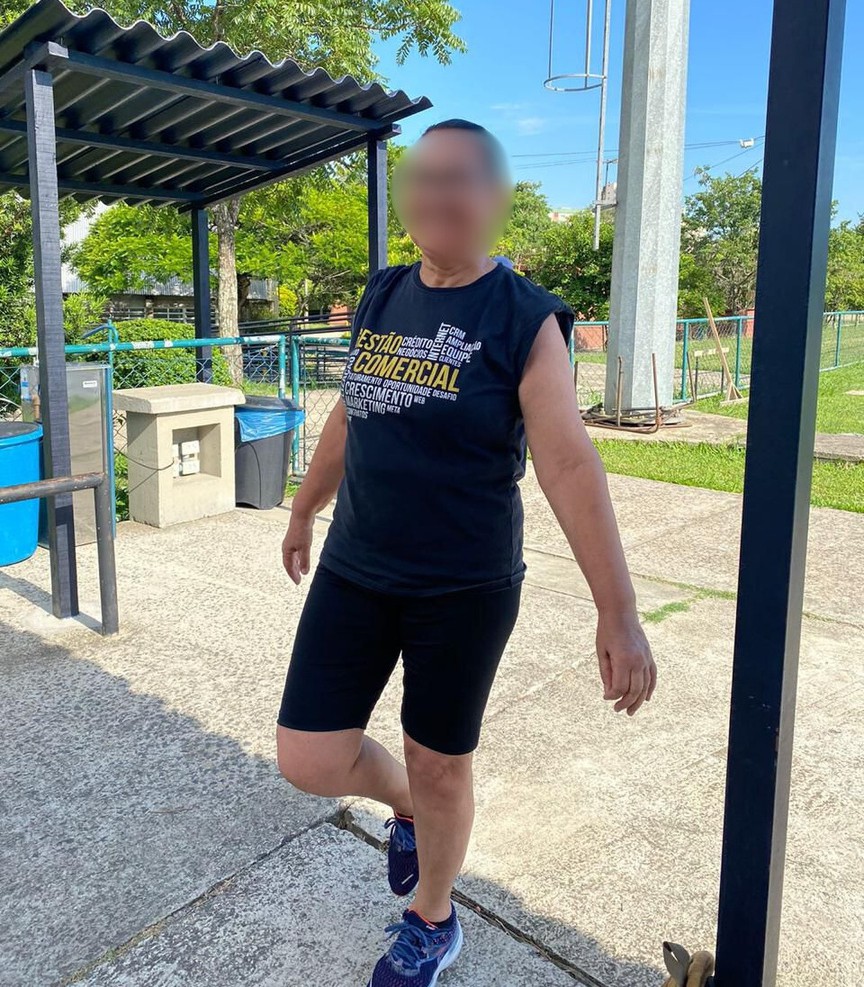


**Unilateral Balance Setup:** standing position on one leg,

hands free or lightly holding a support if needed.

**Execution:** maintaining single-leg stance with upright posture and controlled body alignment.

**Intensity adjustment: -**

**Push-up**

**Setup:** hands placed on the bars, body aligned, feet on the ground.

**Execution:** Push your body away from the bar through elbow extension and shoulder flexion.

**Intensity adjustment:** bar height and body inclination relative to the ground, pace of movement, or range of motion

| **Table S2.** Baseline demographic and clinical characteristics of the participants who completed the protocol twice per week (CT2) or four times per week (CT4) with an attendance rate of at least 80%. | | | | |  |
| --- | --- | --- | --- | --- | --- |
| **Variables** | **CT2 (n=36)** |  | **CT4 (n=27)** | ***P* value** |  |
| Men/Women, n | 16/20 |  | 13/14 | 0.770 |  |
| Age, years | 63 ± 7 |  | 64 ± 6 | 0.594 |  |
| Body weight, kg | 77 ± 2 |  | 77 ± 2 | 0.089 |  |
| Height, cm | 164 ± 8 |  | 165 ± 10 | 0.587 |  |
| BMI, kg/m² | 29 ± 1 |  | 29 ± 1 | 0.174 |  |
| Waist, cm | 100 ± 2 |  | 98 ± 2 | **<0.001** |  |
| **Ethnicity, n(%)** |  |  |  |  |  |
| White | 27 (75) |  | 19 (70) | 0.083 |  |
| Black | 3 (8) |  | 7 (26) |  |  |
| Indigenous | 6 (17) |  | 1 (4) |  |  |
| **Anti-hypertensive medications, n(%)** | |  |  |  |  |
| Diuretics | 19 (53) |  | 13 (48) | 0.716 |  |
| β blockers | 12 (33) |  | 6 (22) | 0.334 |  |
| ARBs | 19 (53) |  | 11 (41) | 0.343 |  |
| CCB | 12 (33) |  | 6 (22) | 0.334 |  |
| ACEI | 7 (19) |  | 13 (48)* | **0.015** |  |
| Combined Therapy | 23 (62) |  | 17 (63) | 0.939 |  |
| **Comorbidities, n(%)** |  |  |  |  |  |
| Diabetes mellitus | 7 (19) |  | 5 (19) | 0.926 |  |
| Hypercholesterolemia | 21 (57) |  | 13 (48) | 0.422 |  |
| Values are mean ± SD. ARBs: Angiotensin II receptor blockers; CCB: Calcium channel blockers; ACEI: Angiotensin converting enzyme inhibitors; Bold p-values indicate significant results (*P* < 0.05). *CT2 different from CT4 (P < 0.05). | | | | |  |
|  |  |  |  |  |  |
